# COLLABORATION WITH FIRST RESPONDERS IN R3: THE CRITICAL ROLE OF COMMUNITY PARTNERS IN HOUSING WITH SERVICES

**DOI:** 10.1093/geroni/igz038.2855

**Published:** 2019-11-08

**Authors:** Pamela Nadash, Edward Miller, Elizabeth Simpson, Natalie Shellito

**Affiliations:** University of Massachusetts Boston, Boston, Massachusetts, United States

## Abstract

Because seniors represent a rising proportion of Emergency Medical Services (EMS) provider activity, there is a growing focus on determining how EMS providers can better serve the aging population. Multifamily senior housing properties benefit from reductions in resident turnover; EMS providers aim to improve community health and minimize unnecessary and low-priority callouts. Developing partnerships between the two service sectors can help both meet their individual goals while reducing health system costs. Drawing on experience with the R3 initiative, this study describes how a partnership between EMS providers and supported housing sites has led to reductions in ambulance transfers to hospitals and reinforced falls reduction programs within senior housing. The R3 program represented an effort to work closely with EMS providers and, by doing so, provides an example of how collaboration between the two sectors can work to the advantage of both parties by identifying residents who would benefit from intervention.

